# Quantitative volcanic susceptibility analysis of Lanzarote and Chinijo Islands based on kernel density estimation via a linear diffusion process

**DOI:** 10.1038/srep27381

**Published:** 2016-06-06

**Authors:** I. Galindo, M. C. Romero, N. Sánchez, J. M. Morales

**Affiliations:** 1 Spanish Geological Survey, Unit of Canary Islands, Alonso Alvarado, 43, 2°A, 35003, Las Palmas de Gran Canaria, Spain; 2 University of La Laguna, Department of Geography, 38207, La Laguna. Tenerife, Spain; 3 University of Las Palmas de Gran Canaria, Faculty of Teacher Training, Special Didactics Department, 35004, Las Palmas de Gran Canaria, Spain

## Abstract

Risk management stakeholders in high-populated volcanic islands should be provided with the latest high-quality volcanic information. We present here the first volcanic susceptibility map of Lanzarote and Chinijo Islands and their submarine flanks based on updated chronostratigraphical and volcano structural data, as well as on the geomorphological analysis of the bathymetric data of the submarine flanks. The role of the structural elements in the volcanic susceptibility analysis has been reviewed: vents have been considered since they indicate where previous eruptions took place; eruptive fissures provide information about the stress field as they are the superficial expression of the dyke conduit; eroded dykes have been discarded since they are single non-feeder dykes intruded in deep parts of Miocene-Pliocene volcanic edifices; main faults have been taken into account only in those cases where they could modified the superficial movement of magma. The application of kernel density estimation via a linear diffusion process for the volcanic susceptibility assessment has been applied successfully to Lanzarote and could be applied to other fissure volcanic fields worldwide since the results provide information about the probable area where an eruption could take place but also about the main direction of the probable volcanic fissures.

Oceanic volcanic islands should address the analysis of volcanic risk in order to develop emergency plans before unrest, since they are geographically isolated territories and crisis management may be complex. Scientists should provide stakeholders with high-quality volcanological, volcanic susceptibility, hazard and risk maps. Volcanic long-term hazard analysis must be the first step, starting by volcanological studies that allow the creation of a volcanic susceptibility map showing the probability of new volcanic vent opening^1^.

Volcanic susceptibility maps must be based on structural data as volcanic vents, eruptive fissures, dykes and faults^2^. However, a common limitation of this analysis relies on the lack of well-detailed knowledge of the volcano structure^2^. Thus, susceptibility analysis must be based on reviewed structural datasets gathered with volcanological criteria (eg. refs 2, 3, 4, 5), rather than in datasets extracted from geological maps that are based only on geological stratigraphic criteria. That is the case of the previous Lanzarote susceptibility map^6^ created to test a new GIS plugin for estimating volcanic susceptibility.

Oceanic volcanic islands are part of huge volcanic edifices that rise from the sea bottom. The emerged part in most oceanic islands represents less than 10% of the total volume of the volcanic island edifice^7^. So, underwater structures can also provide valuable information for the susceptibility analysis. A good example of the improvement in the results of the susceptibility analysis taking into account data from the submerged part of the edifice is the susceptibility map of El Hierro Island^5^. Thus, submarine data are also important and should be always considered when approaching the probability of new vents opening in insular territories.

Fissure volcanic fields are characterized by a linear distribution of structures. This uniqueness must be considered in the susceptibility analysis. Previous studies usually approached the susceptibility analysis by applying probability density functions (PDFs) by core features or Kernel of Gauss type^1^,^2^,^3^,^4^,^5^,^8^,^9^,^10^,^11^). These algorithms have bandwidth acting in one direction of space providing a radially symmetric kernel function. This core function has little local adaptation and an enormous sensitivity to data that are outside the range of values (outliers), with a clear tendency to flatten highs and lows of the function, and do not collect this uniqueness. A better fit with the linear distribution of volcanic fields is obtained by applying a two bandwidth Kernel function^12^,^13^ by a method of bandwidth selector^14^,^15^ with adaptive directional anisotropy data.

The geology of Lanzarote is characterized by the existence of three old massifs mainly outcropping to the North and South of the Island and a Quaternary fissure volcanic field, which also define the islets volcanism (Fig. 1A). The Old Massifs are the remnants of Miocene to Pliocene shield volcanoes (15.5–3.8 Ma^16^). After the formation of the Old Massifs, the magmatic activity ceased and an intense period of erosion followed. Later on, volcanic activity resumed in the central sector (2.7 Ma^16^).

Two historical eruptions took place in the central area of the island (Fig. 1A): the 1730–36 Timanfaya eruption and the 1824 triple eruption^17^. Both were multiple-fissure type eruptions but quite different in size. Timanfaya constitutes the highest magnitude eruptive process occurred in historical times in the Canaries taking into account the total number of eruption days (79% of the total amount of days with volcanic activity in the archipelago), and the area total covered by volcanic material (73% of the total area affected by historical eruptions in the archipelago). It lasted nearly 6 years and formed hundreds of vents aligned along a 13 km eruptive fissure, from where lava flows that covered one-third of the island were issued^17^,^18^,^19^,^20^. By contrast, during the 1824 eruption three small eruptive fissures were formed emitting few pyroclastics and small lava flows with a length in the order of hundred meters^17^,^18^.

The aim of this work is to provide the first long-term volcanic susceptibility map of Lanzarote volcanic edifice from the sea bottom applying kernel density estimation via a linear diffusion process. The input data derive from a chronostratigraphic revision of the Quaternary volcanism as well as the detailed mapping of volcanic vents and faults. Additionally, new vent alignments have been inferred and the role of volcanic vents and alignments, dykes and faults have been reviewed in this volcanic susceptibility analysis.

## Results

### Chronostratigraphic revision

The lack of chronological data is one of the main problems when facing susceptibility analysis, particularly in those volcanic areas with few historical eruptions and discontinuous activity during a long period of time. These data are important to assign an absolute or, at least, a relative age to the different deposits and structures. In Lanzarote Island, to the scarcity of data and the controversial methods used, it must be added the problem of disagreement between authors in some cases and the introduction of transcription errors and terminological confusion in other cases.

**Figure 1:**
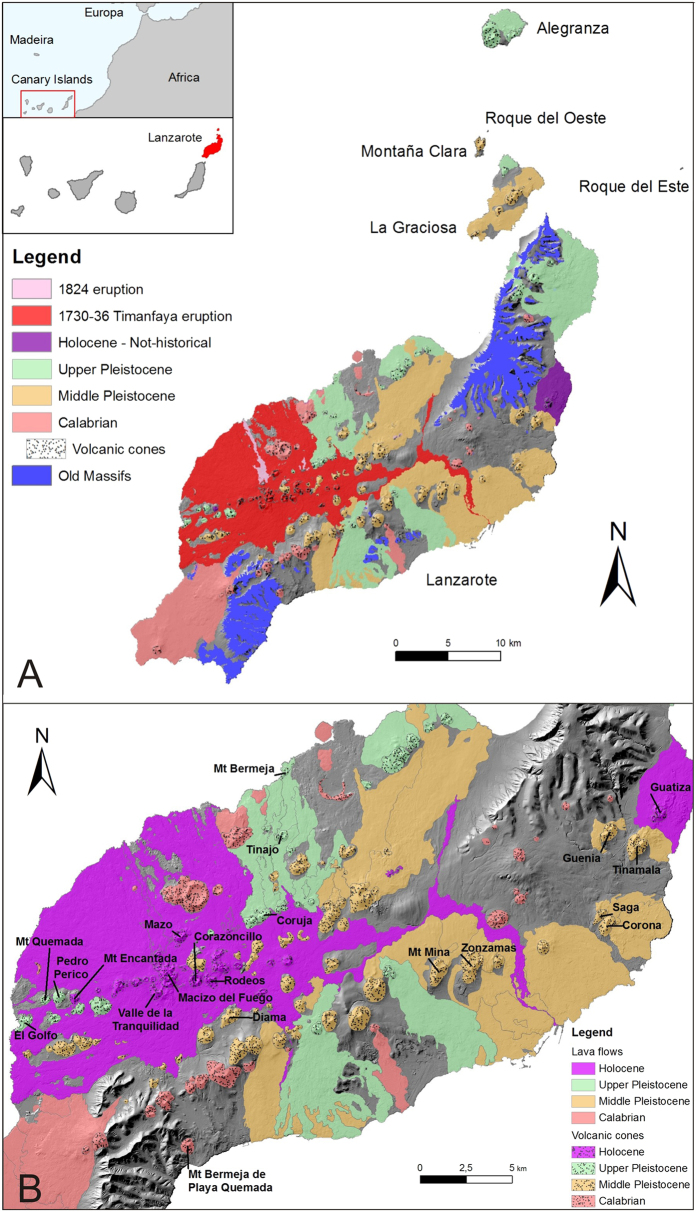
(**A**) Old massifs and quaternary volcanic deposits of Lanzarote Island and Chinijo Islets (Montaña Clara, Alegranza, La Graciosa, Roque del Este and Roque del Oeste). Quaternary lava flows not clearly related with an emission center and sedimentary deposits have not been represented. The inset maps display the geographical setting. (**B**) Detail of the quaternary volcanic deposits in the central area of Lanzarote. Names in the map refer to volcanic groups in which the relative age has been modified. Maps created with ESRI ArcGIS 9.3.1 (http://www.esri.es/es/productos/arcgis/).

That is why the first step in this procedure has been to make a chronostratigraphical revision with the objective of assigns a relative age, adapted to the new Chronostratigraphical Chart (http://www.stratigraphy.org/index.php/ics-chart-timescale), to the outcropping deposits and their associated structures. Therefore, the chronostratigraphy of the Quaternary deposits of Lanzarote and Chinijo Islands has been updated for this work. Previous geochronological data^16^,^21^,^22^,^23^,^24^ have been adapted according to the new international geochronological chart (see supplementary Table S1). Revision of previous data, mapping of cinder cones and lava flows and field observations allow obtaining a new cartography of cinder cones and lava flows for the Lanzarote and Chinijo Islands quaternary deposits (Fig. 1B). Changes introduced in the chronostratigraphy are detailed in the supplementary material (SI Discussion).

### Volcano-tectonic structures and their role in the susceptibility analysis

**Figure 2:**
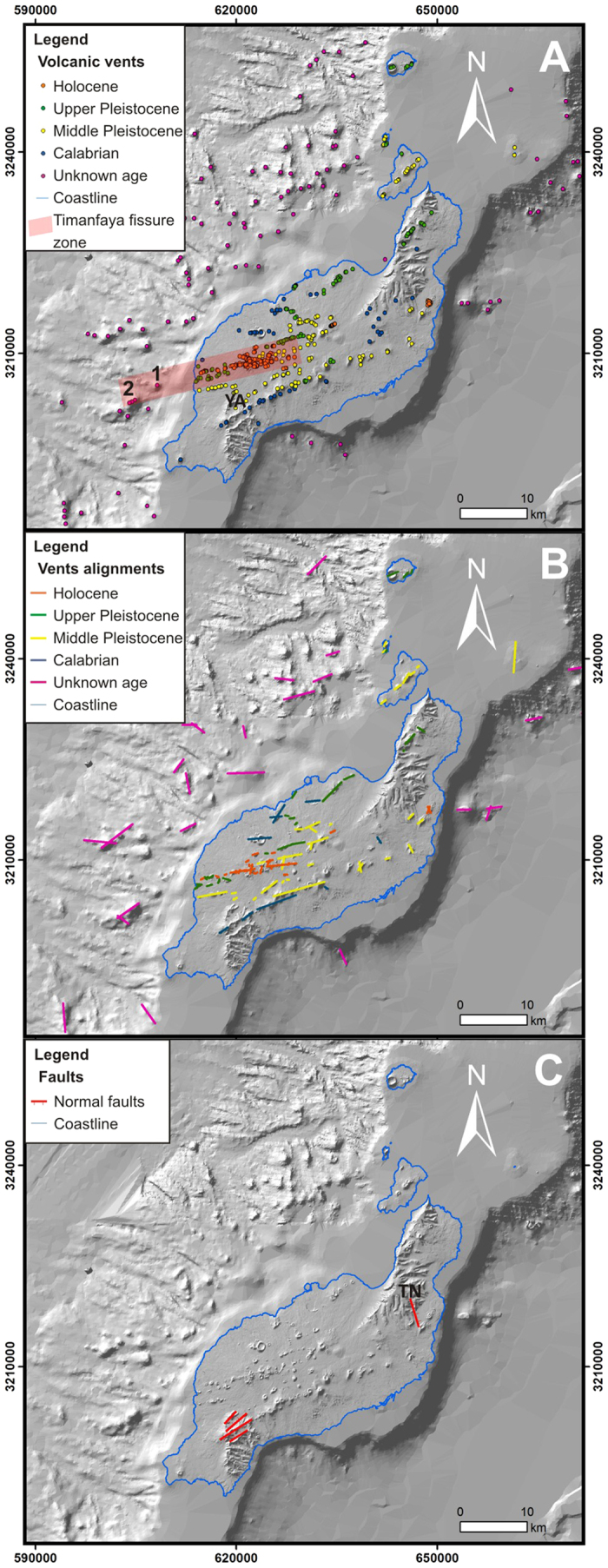
Structural data of Lanzarote and Chinijo Islands considered for the susceptibility analysis. (**A**) Volcanic vents; 1 and 2 indicate the location of submarine volcanic cones from the Timanfaya eruption; YA, location of Yaiza village. (**B**) Vent alignments. (**C**) Faults; TN, location of Teneguime valley. Maps created with Matlab R2007A (http://es.mathworks.com/products/matlab/).

Spatial probability of new vents opening is based on the hypothesis that establishes that future volcanic vents will be formed near previous ones^9^,^25^, since we assume that their distribution is controlled by a similar stress field, indicating where the crust has the favourable stress conditions for the intrusion of magma up to the surface. Thus, location of volcanic vents is a key element in the susceptibility analysis. In Lanzarote, vents are concentrated largely in the central part and western submarine flank of the Island (Fig. 2). In the eastern flank, however, they are very scarce. A total of 613 volcanic vents have been mapped: 476 in Lanzarote Island, 28 in the Chinijo Islets and 109 underwater. We have mapped more than twice the number of vents with respect to the identified in previous works^6^,^26^, plus the Chinijo Islands vents and the submarine volcanic cones.

Regarding the 244 superficial Holocene vents, most of them (82%) are related to the 1730–36 eruption, being 13% from the 1824 eruption and 5% are not-historical. The remaining superficial vents were formed during the Upper Pleistocene (20%), Middle Pleistocene (23%) and Calabrian (9%). The age of the submarine vents is unknown; except for the Roque del Este vents that are Middle Pleistocene just like the deposits outcropping on the islet. In addition, there are some cones underwater related to the 1730–36 eruption. The existence of a submarine phase was previously mentioned by other authors based on historical chronicles^17^,^18^,^19^,^27^), saying: “By the end of June 1731, the sea banks on the occidental side of the island were covered with a vast amount of dying fish from the most diverse sorts, some of them in shapes never seen before. Towards the NW of Yaisa one could see a huge cloud of smoke and flames coming out of the sea accompanied by tremendous detonations, and all around the Rubicon Sea, I mean, over the west coast, the same could be seen, floating around fish and pumice stones”.

Thus, we can presume that some of the volcanic cones mapped on the western coast of Yaiza could have been formed during the first year of eruption. If we observe this area an isolated submarine vent is located at the prolongation of the Timanfaya main fissure, around 6 km from the coast (Fig. 2A,B). The historical documents suggest the existence of more than one vent, so offset vents to the SW might be also related to the eruption. The summits of these cones are around 500–1000 m below sea level, suggesting intensive erosion after the eruption.

In addition to vents, vent alignments must be considered for the susceptibility analysis since they represent the eruptive fissures. Although they do not provide the location of a punctual vent, they inform about the stress field, since they are basically pure open fractures driven by magmatic overpressure, being perpendicular to the minimum compressive principal stress σ_3_^28^. Along the studied area we have inferred 90 subaerial and 26 submarine vent alignments (Fig. 2B). Most of these alignments are NE-SW or ENE-WSW in trends. The length of these structures ranges from 150 m to 8 km, with a mean value of 1.7 km. Only few are of Calabrian age. The number of Holocene and Pleistocene vent alignments is similar.

Dykes have been usually considered in susceptibility analysis. However, a very low weight (0.02–0.15) is always assigned to them^2^,^5^,^6^. They are generally formed in the same regional stress field, but non-feeders become arrested because the local stress field, related to local mechanical properties, is unfavourable^29^. When dykes outcrop in eroded parts of volcanic edifices it may be considered that most of them did not reach the surface^30^,^31^, and hence cannot be related to volcanic fissures. This fact is also supported by the high number of unrest volcanic periods characterized by magma intrusion that never leads to eruptions (eg. refs 32,33). In addition, dykes intruding the same edifice may have been injected under different local stress conditions, since the stress field may change with time and depth^34^,^35^. Dyke planes, as discontinuities in the crust, can also be used as easy pathways for new magma intrusions, although the percentage of multiple dykes in fissure volcanic fields is always very low in comparison with simple dykes^26^,^36^. Another fact is that dykes mapping at a 1:25.000 scale are usually exaggerated (since dykes are usually a few meters thick). Besides, the cartographic expression of dykes displays a lot of intersections representing the main directions of dyke swarms. In Lanzarote, dykes are mainly located in the eroded remnants of the Miocene-Pliocene Old Massifs and are not related to the Holocene volcanic deposits, well preserved in Lanzarote and clearly resting unconformably over the eroded Old Massifs deposits. Field observations confirmed that these dykes do not show feeder’s typical characteristics^37^. Marinoni and Pasquarè^26^ identified only a multiple dyke from the 273 studied in Lanzarote. Thus, only a 0.4% of the injected magma followed previous dykes in their ascent. Taking into account these observations to count dykes in our study will result in an artificial increase of the local probability, that statistically is correct but have no sense for the susceptibility analysis. For all these reasons, we have decided to exclude dykes in this study, and propose to do not use them except in those cases where they are feeders and the related eruptive fissure is not mapped.

Previous faults can also capture magma intrusions and modified dyke direction when the magma propagates through the fault plane^2^,^37^,^38^. For example, a clear example of an eruptive fissure changing in direction when the feeder dyke propagates through a fault plane has been reported in the old southern massif 39. Field evidences suggest that small fractures and faults do not affect the main propagation of the feeder dyke^37^. Thus, only five well developed faults have been included in the faults dataset (Fig. 2C). In the southern old massif, four parallel fault planes form a graben structure, one of which has a well-developed breccia and shows slickensides. Along the Tenegüime valley there is another fault^26^ that is consistent with field observations.

### The susceptibility map of Lanzarote and Chinijo Islands

**Figure 3:**
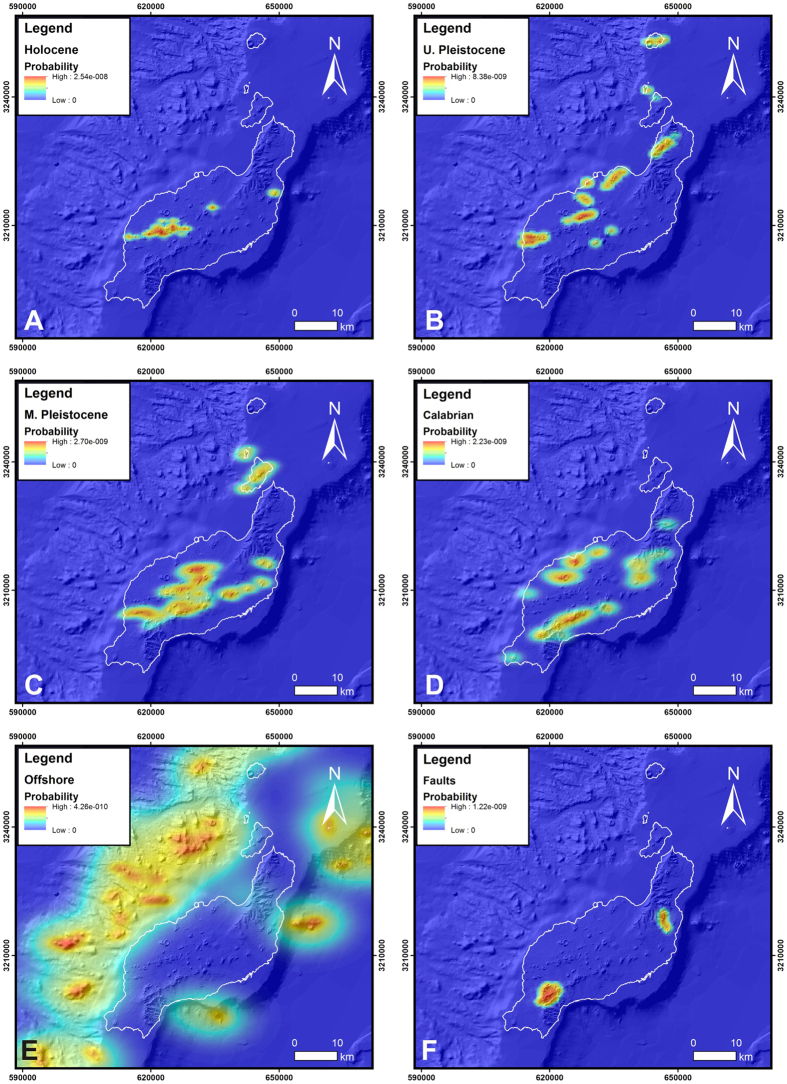
PDF of Holocene vents and vent alignments (**A**), Upper Pleistocene vents and vent alignments (**B**), Middle Pleistocene vents and vent alignments (**C**), Calabrian vents and vent alignments (**D**), submarine vents and vent alignments (**E**) and, faults (**F**). Maps created with Matlab R2007A (http://es.mathworks.com/products/matlab/).

The reliability of the Botev´s algorithm^15^ to estimate the probability of vent opening in Lanzarote has been tested successfully using all the structural data except those formed during the last 1824 eruption (see supplementary Discussion S1). To generate the volcanic susceptibility map Probability Density Function (PDF) maps have been created using structural data. These structural data have been divided into 11 datasets regarding the type of data and their age, obtaining 4 PDF for subaerial vents for the Holocene, Upper Pleistocene, Middle Pleistocene and Calabrian, respectively; 4 PDF for vent alignments for the same Series, 2 PDF for submarine vents and alignments, and a PDF for faults. In order to simplify the number of Probability Density Function (PDF) maps, subaerial vents and vents alignment of the same age as well as the submarine ones have been considered in the same dataset finally obtaining 5 PDF (Fig. 3A–E). These PDF together with the faults PDF (Fig. 3F) constitute the basis for the susceptibility map.

**Figure 4:**
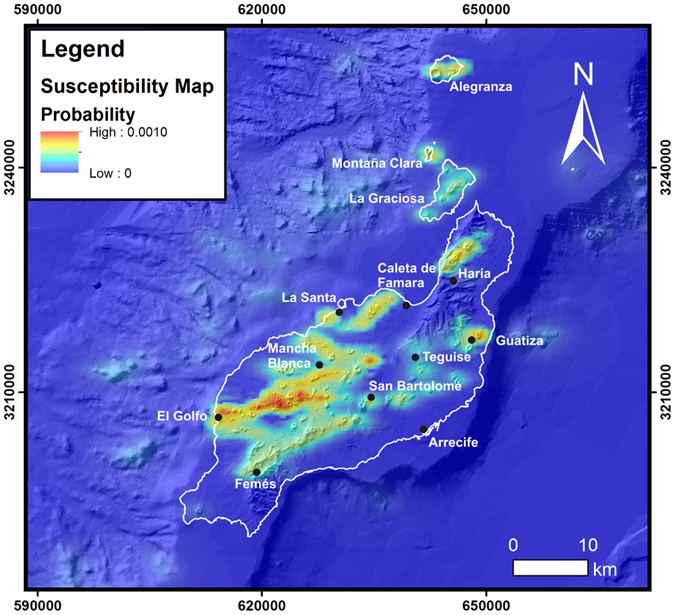
Volcanic susceptibility map of Lanzarote and Chinijo Islands. Maps created with Matlab R2007A (http://es.mathworks.com/products/matlab/).

The susceptibility map of Lanzarote and Chinijo Islands (Fig. 4) shows that high susceptibility values are disposed in roughly elongated areas with directions ranging between NE-SW and ESE-WSW. The highest susceptibility zone, with values up to 0.0010, is located in the central-western sector of the island, between El Golfo and San Bartolomé (Fig. 4). This area is elongated in an ENE-WSW direction and extends far from the coast. It is clearly associated to the high concentration of vents and vent alignments from the 1730–36 eruption. In this area is also located the southwestern fissure of the 1824 triple eruption. Relatively high susceptibility values are also found south and east of Mancha Blanca due to the presence of the other two eruptive fissures formed during the 1824 eruption. Similar values are located in the Guatiza area related to the Holocene Guatiza calderas volcanic group and the presence of the Tenegüime fault.

Areas with medium susceptibility values are developed to the north of Haría, west of Caleta de Famara and between La Santa and Mancha Blanca (Fig. 4). The first is associated to the Upper Pleistocene volcanic groups of Monte Corona and Helechos and the rest to the presence of Upper Pleistocene volcanism. All these areas include part of the coastal submarine area. An anomaly characterized by susceptibility intermediate values is located between San Bartolomé and Femés (Fig. 4), mainly related to the coexistence of high probability values for faults (Fig. 3F) and for Middle Pleistocene and Calabrian vents and alignments (Fig. 3C,D).

The lowest susceptibility values appear in the Chinijo Islets, except for Alegranza Islet, and in the submarine part. The western submerged flank is also characterized by low susceptibility values displaying scattered zones that are linked to a greater concentration of vents in the underwater flanks. The susceptibility values of the underwater eastern flank are very low.

Comparing these results with the Lanzarote previous susceptibility map^11^, the highest susceptibility values are in the same order of magnitude and both are related to Holocene eruptions. Nevertheless, high and medium susceptibility values in our map are not restricted to these areas, but widely scattered around the island. In contrast with previous volcanic susceptibility maps for Lanzarote^6^ or other volcanic areas^1^,^2^,^3^,^4^,^5^, based on a Gaussian kernel and a spaced grid greater than 200 m, the use of a lower cell size and the application of the Botev’s algorithm produce constrained elongated areas adapted to the vent alignments trend rather than to broadly distributed areas. Thus, it provides information about the probable direction of future eruptive fissures.

The low values of susceptibility found in the area between Teguise-San Bartolomé-Arrecife seem to coincide with positive density and magnetic anomalies that have been related to the presence of an intrusive body^40^,^41^. The presence of such intrusive body could make difficult the ascent of magma to the surface explaining the lack of eruptions in this area since the Pleistocene. A similar effect has been recently described using tomographic technics in El Hierro Island during the submarine eruption in 2011–2012^42^.

## Conclusions

Volcanic susceptibility analysis must be based on reviewed volcano-structural data at a minimum scale of 1:5.000, since the improvement of the datasets results in a more reliable susceptibility map. Structural elements should include vents, eruptive fissures, feeder dykes and main faults. Using non-feeder dykes in the volcanic susceptibility analysis is not recommendable since they probably were intruded under different stress conditions than the one controlling magma emplacement close to the surface. Information about the submarine structures must be included when dealing with volcanic islands. Although some data are difficult to obtain and the geological knowledge is usually poor, an effort must be done in order to review and collect the necessary good quality data. The Lanzarote and Chinijo Islands volcanic susceptibility map has been based on a volcanological review of the Quaternary deposits and structures, and the geomorphological analysis of the submarines flanks. The number of structures used in the susceptibility analysis has been significantly higher than those in previous works improving the resulting map. Taking into account submarine data also improves the results providing susceptibility information that should be considered previously to the installation of submarine cables for communication purposes or other type of structures (pipes, wind turbines, etc). The volcanic susceptibility in coastal areas, usually with high touristic activity in volcanic islands, is also necessary for risk stakeholders.

The application of a Botev’s algorithm results in a better fit of the model to the spatial distribution of the fissure volcanism of Lanzarote and Chinijo Islands and reduces the effect of outliers. High to medium susceptibility values are constrained to elongated areas that trend in the same direction than the main vent alignments. Ergo, the kernel density estimation via a linear diffusion process method used to estimate the volcanic susceptibility provides information about the stress field, illustrating the probable main directions that new eruptive fissures could have. This method also results in a better adjustment of the probability areas and allows obtaining a better resolution by decreasing the cell size. Accordingly, this method could be applied worldwide in other volcanic fields characterized by fissure type eruptions.

## Method

We have studied the Quaternary volcanic deposits and structures of Lanzarote based on detailed fieldwork, the revision of the geological maps at scales 1:25,000 and 1:100,000^22^,^23^,^24^,^43^,^44^,^45^,^46^,^47^,^48^,^49^,^50^,^51^, the digital geological map^52^, the LIDAR based Digital Terrain Model with 5 m mesh size (Centro Nacional Información Geográfica, http://pnoa.ign.es/coberturalidar), the analysis of orthophotos and vertical aerial photographs at scale 1:5,000 and 1:18,000, respectively; and the bathymetric data provided by REDMIC (Dirección General de Sostenibilidad de la Costa y el Mar, Ministerio de Agricultura, Alimentación y Medio Ambiente, coastal areas at 1 m resolution; Instituto Español de Oceanografía, 50 m resolution) and the Instituto Hidrográfico de la Marina (100 m resolution). Software ArcGIS 9.3 by ESRI© has been used and data have been georeferenced in UTM 28 N-WGS84. Mapping has been made at a scale 1:5,000.

Superficial and undersea volcanic cones have been mapped. Those subaerial have been included in volcanic groups after reviewing the stratigraphic relationships of the deposits. These volcanic groups include deposits probably formed during the same eruption and have been assigned to a stratigraphic Series following the latest proposed international chronostratigraphic chart (http://www.stratigraphy.org/index.php/ics-chart-timescale). Vents have been mapped following Becerril *et al.*^5^ criteria. All spattered cones that are related to underground drainage of lava flows and pseudocraters have been discarded, since they are volcanic structures without structural significance. Vent alignments have been mapped tracing a line between: (1) two vents included in the same volcanic edifice or in a coalescent one with the same weathering degree or; (2) 3 or more vents each distant less than 2 km. For those cases with high density of vents, like the Timanfaya eruption, only two fissures have been defined, prioritizing those fissures defined by the best developed volcanic cones. The age of the volcanic group has been assigned to the vents and vents alignments.

Dykes, faults, proposed photo lineaments and inferred faults have been checked in the field and only those well-developed faults clearly identified in the field have been included in the cartography. To generate the volcanic susceptibility model, PDF maps have been created for the different structural datasets (vents, vent alignments and faults) regarding their age and location. A square cell size of 200 m has been considered. In order to simplify the number of PDF, vents and vents alignment of the same age have been grouped in the same PDF by using equation (1):


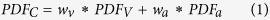


where PDF_c_ is the resulting PDF of the combination of vents and vent alignments, w_v_ is the weight assigned to PDF of vents (PDF_v_) and w_a_ is the weight assigned to the PDF of vents alignments (PDF_a_). w_v_ and w_a_ have been selected attending to the reliability of the data (see supplementary material Table S2). Since vent alignments are based on the cartography of vents, their reliability decreases with the age of the structure, being more reliable the location of the vent. In this manner, we have obtained five PDF of vents and vent alignments: Holocene, Upper Pleistocene, Middle Pleistocene, Calabrian and Submarine.

We have applied the Botev’s method^15^ that uses an adaptive kernel density estimation based on the smoothing properties of linear diffusion processes. This core function fits data where a preliminary data model is not required; hence previous interpretation data of the kernel functions can be avoided. It advantageously provides a considerable reduction of the asymptotic bias and the mean integrated squared error, and a proper fit in the biased edge. This estimator fits very well with the volcanic uniqueness and at the same time reduces the problems of isolated elements (outliers).

The Botev’s algorithm^15^ uses a non-radial kernel function based on a pilot bandwidth selector with two axes (N-S and E-W) with different dimensions. The bandwidths sizes for the Gaussian function of each PDF can be consulted in the supplementary Table S3. Each PDF of the six selected (Fig. 3) have been multiplied by a weight that is based on the age of the data and their influence in the probability of opening a new vent as follows: 0.3 to the Holocene vents and vent alignments, 0.25 to the Upper Pleistocene vents and vent alignments, 0.2 to the submarine vents and vent alignments, 0.15 to the Middle Pleistocene vents and vent alignments, 0.08 to the Calabrian vents and vent alignments and 0.02 to the faults. The susceptibility map results from the application of equation (2):


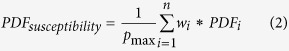


where *p*_*max*_is the maximum probability, *w*_*i*_ is the general weight for each dataset, *PDF*_*i*_ is the Probability Density Function for each dataset, and *N* represents the number of dataset.

## Additional Information

**How to cite this article**: Galindo, I. *et al.* Quantitative volcanic susceptibility analysis of Lanzarote and Chinijo Islands based on kernel density estimation via a linear diffusion process. *Sci. Rep.* **6**, 27381; doi: 10.1038/srep27381 (2016).

## Supplementary Material

Supplementary Information
