# Supplementary material for: Quantitative volcanic susceptibility analysis of Lanzarote and Chinijo Islands based on kernel density estimation via a linear diffusion process
Source: Sci Rep. 2016 Jun 6;6:27381. doi: 10.1038/srep27381 (PMC4893674; doi:10.1038/srep27381)
Supplement: Supplementary Information [file srep27381-s1.pdf]

Supplementary Information

Quantitative volcanic susceptibility analysis of Lanzarote and Chinijo Islands based on kernel density estimation via a linear diffusion process

I. Galindo[1*], M.C. Romero[2], N. Sánchez[1], J.M. Morales[3]

[1] Spanish Geological Survey, Unit of Canary Islands, Alonso Alvarado, 43, 2ºA, 35003, Las Palmas de Gran Canaria, Spain. i.galindo@igme.es; n.sanchez@igme.es

[2] University of La Laguna, Department of Geography, 38207, La Laguna. Tenerife, Spain. mcromero@ull.edu.es

[3] University of Las Palmas de Gran Canaria, Faculty of Teacher Training, Special Didactics Department, 35004, Las Palmas de Gran Canaria, Spain. jmmdef@gmail.com

[*] Corresponding author e-mail: i.galindo@igme.es

SI Discussion

SI Discussion S1: Changes introduced in the chronostratigraphy of Lanzarote

Mazo volcano (Fig. 1B) was traditionally considered as one of the 1730-36 Timanfaya eruption cones (1). However, other authors excluded this cone and also Corazoncillo caldera from the historical period based on paleomagnetic data that are considered as not conclusive in the same paper (2). These changes were included in the 2005 geological map (3), where Mazo is part of an Upper Pleistocene volcanic alignment although no criteria are provided for this age assignment. Mazo volcano is very well preserved and the issued lava flows are covering lavas from the first episodes of the Timanfaya eruption (Fig. 1B). In addition, the revision of the Timanfaya historical documents suggests that Mazo volcano was formed during the first months of the Timanfaya eruption. Based on field observations (1), Corazoncillo caldera, Mt. Rodeos, Valle de la Tranquilidad and Macizo del Fuego are also included as Timanfaya edifices.

Mt. Encantada was previously thought to be of Middle Pleistocene age (3, 4) and was included in the alignment of Pedro Perico and Mt. Quemada (Fig. 1B). Although this cinder cone is very well preserved, it has a high density of fissures probably related to a partial collapse. Fieldwork has revealed that there is a thin layer of reworked lapilli between Mt. Encantada lapilli and the Timanfaya lava flows (Fig. S1A), suggesting only a small hiatus between both eruptions. Thus, this eruption has been considered as Holocene.

The Guatiza calderas are included in the geological map as Holocene, since a marine terrace of abrasion has not yet been fully developed over their lava flows at the coast, and the lava flows seem to be over the Erbanian Holocene marine deposits (5, 6). However, it was considered a volcanic group together with Tinamala and Guenia volcanoes that are worse preserved, more eroded and present calcretes. Thus, Guatiza has been maintained as Holocene and an Upper Pleistocene age has been assigned to Tinamala and Guenia (Fig. 1B).

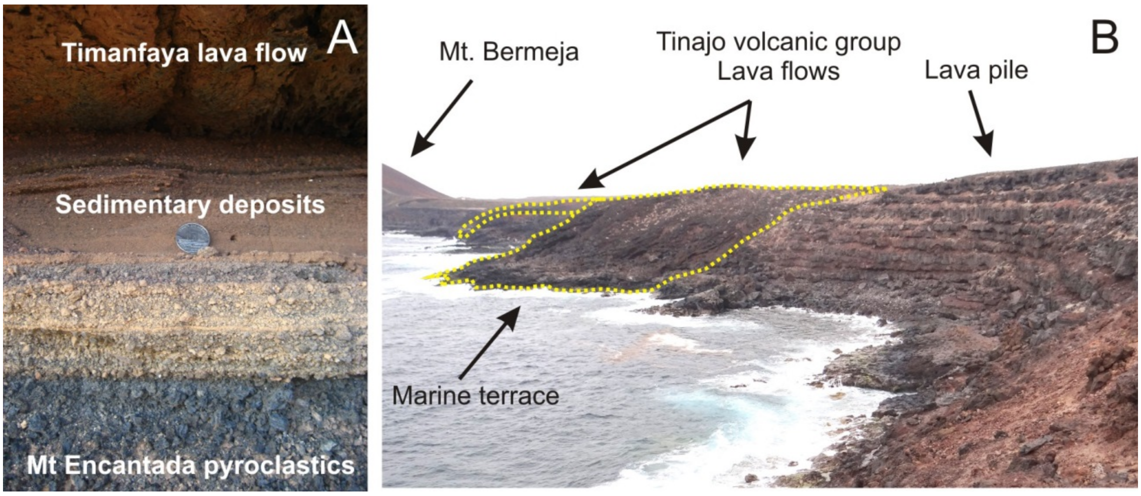

SI Figure S1. Stratigraphic relationships between some deposits of Lanzarote: A) Reworked deposits between Mt. Encantada pyroclastics and a Timanfaya pahohoe lava flow; B) Lava flows from the Tinajo volcanic group volcanic group covering the lava pile deposits that constitute the 30 m high cliff. Observed the marine terrace formed over the Tinajo volcanic group lava flows. Yellow dotted lines show the limits of the Tinajo volcanic group lava flows. See location of cones in Fig. 1 (main manuscript).

El Golfo volcano, the ESE end of a nearly 5 km vent alignment (Fig. 1B), has been assumed to be less than 2 Ma (7) based on Abdel-Monem et al. (8). Here there is a terminological confusion and a transcription error since this paper refers to El Golfo edifice in El Hierro Island and provides an age earlier than 0.2 Ma. Based on the geomorphological criteria and the absence of calcretes, we have considered an Upper Pleistocene age for this volcanic group, and also for the Pedro Perico volcanic set, deposits that are stratigraphically over the El Golfo volcanic group deposits.

Tinajo and Coruja volcanic sets were previously thought to be of Middle Pleistocene age (9, 10) but have been included in the Upper Pleistocene since their lava flows are covering the coastal cliff and their deposits are higher stratigraphically than those of the Bermeja volcanic group that is of Upper Pleistocene age (Fig. S1B). A marine terrace of abrasion has been developed over Tinajo lava flows as well as over the Bermeja deposits, but not over the Coruja deposits, which has a fossil beach under its lava flows. Thus, Coruja is younger than Bermeja and Tinajo.

In addition, we have changed the Series of Mt. Zonzamas and Mt. Corona volcanic groups (Fig. 1B) from the Lower (9, 10) to the Middle Pleistocene since they have been dated in 710 and 770 ka (Table S1). Crosscutting relationships of Mt. Mina, Mt. Saga

and Zonzamas Caldera with the El Jable sand dunes and Mt. Zonzamas and Mt. Corona deposits, as well as the degree of weathering, suggest that they were also formed during the Middle Pleistocene.

Mt. Bermeja de Playa Quemada (Fig. 1B) is Pliocene in the geological map at a scale 1:100,000 (3) and Lower Pleistocene at a scale 1:25,000 (11). Its deposits are laying over a Tortonian marine terrace, but the weathering degree is similar to the Calabrian ones. Therefore, we have included it in this age.

Finally, the geological map (3, 9) includes two vents that are in fact sand dunes covered by fallout deposits (1, 12). We have verified that these lapilli deposits are fallout deposits from a near volcanic group, so we have eliminated these vents.

SI Discussion S2: Test of Botev´s algorithm

The reliability of the Botev´s algorithm (13) to estimate the probability of vent opening in Lanzarote have been tested successfully using all the structural data except those formed during the last 1824 eruption (Fig. S2). The results show that the 1824 vents and eruptive fissures were formed in areas of medium to relatively high susceptibility values, except for the northeastern fissure that occurred in a relatively low susceptibility area. None of the 1824 vents are in the lowest susceptibility area. These results allow concluding that the Botev´s algorithm is a good method to estimate the volcanic susceptibility.

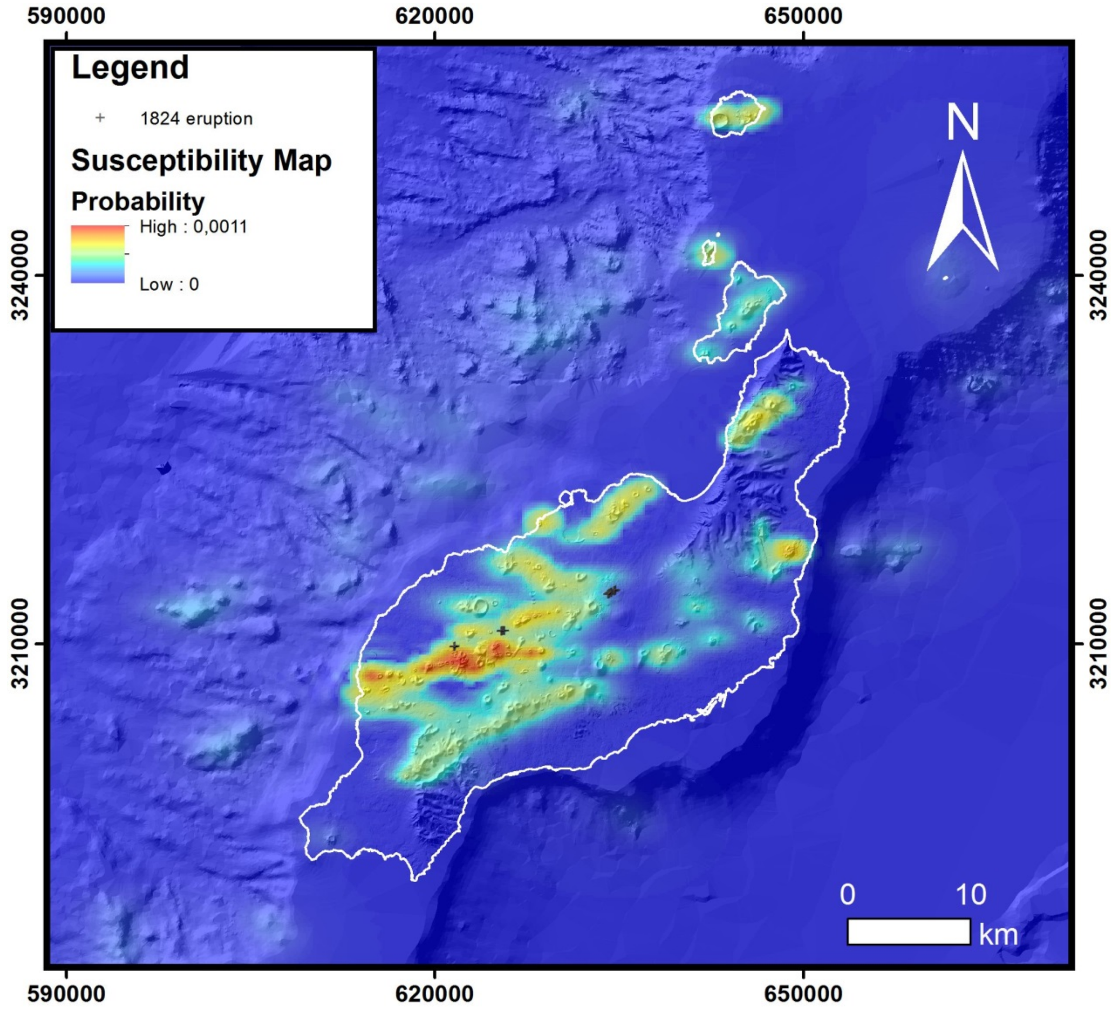

Figure S21. Volcanic susceptibility map of Lanzarote and Chinijo Islands constructed with data previous to the last volcanic eruption in Lanzarote occurred in 1824. Map created with Matlab R2007A (http://es.mathworks.com/products/matlab/).

SI Table Captions:

SI Table S1. Synthesis of the K/Ar ages published for the Quaternary volcanic deposits of Lanzarote Island.

<table>
<tr><th>Age</th><th>Name</th><th colspan="2">Age (ka)</th><th>Reference</th></tr>
<tr><td rowspan="2">Upper Pleistocene</td><td>Mt. Corona</td><td>21</td><td>± 6.5</td><td rowspan="2">(14)</td></tr>
<tr><td>Los Helechos</td><td>91</td><td>± 2</td></tr>
<tr><td rowspan="6">Middle Pleistocene</td><td>Mt. Guatisea</td><td>240</td><td>± 140</td><td rowspan="3">(15)</td></tr>
<tr><td rowspan="2">Mt. Tahiche</td><td>350</td><td>± 60</td></tr>
<tr><td>630</td><td>± 70</td></tr>
<tr><td>Mt. deZonzamas</td><td>710</td><td>± 70</td><td>(16)</td></tr>
<tr><td>Mt. Tinajo</td><td>730</td><td>± 100</td><td>(15)</td></tr>
<tr><td>Mt. Corona</td><td>770</td><td>± 80</td><td>(5)</td></tr>
<tr><td rowspan="9">Calabrian</td><td>Atalaya de Femés</td><td>920</td><td>± 300</td><td rowspan="2">(15)</td></tr>
<tr><td rowspan="3">Mt. Tinasoria</td><td>990</td><td>± 200</td></tr>
<tr><td>1110</td><td>± 70</td><td rowspan="2">(3)</td></tr>
<tr><td>1130</td><td>± 70</td></tr>
<tr><td>Mt. Bermeja de Guime</td><td>1200</td><td>± 100</td><td>(15)</td></tr>
<tr><td>Guanapay</td><td>1200</td><td>± 240</td><td>(15)</td></tr>
<tr><td>Los Valles</td><td>1340</td><td>± 40</td><td>(9)</td></tr>
<tr><td>Pre-Los Valles</td><td>1410</td><td>± 80</td><td rowspan="2">(15)</td></tr>
<tr><td>Mt. del Valle del Palomo</td><td>1800</td><td>± 300</td></tr>
</table>

SI Table S2. Weights assigned to each dataset to calculate the PDF maps: w_v, weight assigned to datasets of vents; w_a, weight assigned to datasets of vents alignments and; w_i, weight assigned to each dataset considered for the susceptibility estimation.

Datasets	w_v	w_a	w_i
Holocene	0.5	0.5	0.30
Upper Pleistocene	0.4	0.6	0.25
Middle Pleistocene	0.3	0.7	0.15
Calabrian	0.2	0.8	0.08
Submarine	0.2	0.8	0.20
Faults	-	-	0.02

SI Table S3. Size and relationship of N-S and E-W bandwidths for the Gaussian function for each PDF.

		Holocene	Upper Pleistocene	Middle Pleistocene	Calabrian	Submarine	Faults
N-S Bandwidth (m)	Vents	387	650	1291	1432	4679	375
	Vents alignments	429	516	657	437	1289	
E-W Bandwidth (m)	Vents	241	423	645	786	3367	386
	Vents alignments	207	304	324	231	780	
Bandwidth relationship	Vents	0.5	0.6	0.7	0.8	0.8	0.97
	Vents alignments	0.5	0.4	0.3	0.2	0.2	

References

1. Romero, C. El relieve de Lanzarote (Servicio de publicaciones del Cabildo de Lanzarote, 2003).
2. Carracedo, J.C., Rodríguez Badiola, E. & Soler, V. Aspectos volcanológicos y estructurales. Evolución petrológica e implicaciones en riesgo volcánico de la erupción de 1730 en Lanzarote. Islas Canarias. Estud. Geol. 46, 25-55 (1990).
3. IGME. Mapa geológico de España escala 1:100.000, 88, Lanzarote. (Instituto Geológico y Minero de España, Madrid, 2005).
4. IGME. Mapa geológico de España escala 1:25.000, Isla de Lanzarote, 1081 II, Yaiza (Instituto Geológico y Minero de España, Madrid, 2004).
5. IGME. Mapa geológico de España escala 1:25.000, Isla de Lanzarote, 1.082 I, Guatiza (Instituto Geológico y Minero de España, Madrid, 2004).
6. IGME. Mapa geológico de España escala 1:25.000, Isla de Lanzarote, 1.080 II, Haría (Instituto Geológico y Minero de España, Madrid, 2004).
7. Pedrazzi, D., Martí, J. & Geyer, A. Stratigraphy, sedimentology and eruptive mechanisms in the tuff cone of El Golfo (Lanzarote, Canary Islands). Bull. Volcanol. 75, 1-17 (2013).
8. Abdel-Monem, A., Watkins, N. & Gast, P. W. Potassium-argon ages, volcanic stratigraphy, and geomagnetic polarity history of the Canary Islands; Tenerife, La Palma and Hierro. Am. J. Sci. 272, 805-825 (1972).
9. IGME. Mapa geológico de España escala 1:25.000, Isla de Lanzarote, 1.082 IV, Teguise (IGME, Madrid, 2004).
10. IGME. Mapa geológico de España escala 1:25.000, Isla de Lanzarote, 1.081 I, Tinajo (Instituto Geológico y Minero de España, Madrid, 2004).
11. IGME. Mapa geológico de España escala 1:25.000, Isla de Lanzarote, 1081 I-IV, Femés (Instituto Geológico y Minero de España, Madrid, 2004).

12. Meco, et al. Paleoclimatologia del Neógeno en las Islas Canarias: Geliense, Pleistoceno y Holoceno (Ministerio de Medio Ambiente, 2006).
13. Botev, Z.I., Grotowski, J.F. & Kroese, D.P. Kernel density estimation via diffusion. Ann. Stat. 38, 2916-2957 (2010).
14. Carracedo, J. C. et al. La erupción y el tubo volcánico del Volcán Corona (Lanzarote, Islas Canarias). Estud. Geol. 59, 277-302 (2003).
15. Coello J. et al. Evolution of the eastem volcanic ridge of the Canary Islands based on new K-Ar data. J. Volcanol. Geotherm. Res. 53, 251-274 (1992).
16. 16 IGME. Mapa geológico de España escala 1:25.000, Isla de Lanzarote, 1.082 II-IV, Arrecife (Instituto Geológico y Minero de España, Madrid, 2004).
